# Supplementary material for: Twelve-year clinical trajectories of multimorbidity in a population of older adults
Source: Nat Commun. 2020 Jun 26;11:3223. doi: 10.1038/s41467-020-16780-x (PMC7320143; doi:10.1038/s41467-020-16780-x)
Supplement: Supplementary file 1 — Supplementary Information [file 41467_2020_16780_MOESM1_ESM.pdf]

Supplementary tables and figures

Twelve-year clinical trajectories of multimorbidity in a population of older adults

Davide L Vetrano et al.

Nature Communications 2020

7 Supplementary tables

15 Supplementary figures

Supplementary table 1. Sample characteristics (weighted means and proportions) by clusters of multimorbidity over time.

Abbreviations: MMSE = Mini Mental State Examination. Weighted means and proportions have been obtained by the membership matrix.

	Baseline N = 2931						6 years N = 1716						12 years N = 1016					
	Psych. and respiratory diseases	Heart diseases	Eye diseases and cancer	Cognitive and sensory impairment	Respiratory and MSK diseases	Unspecific group	Heart and vascular diseases	Heart diseases and cognitive impairment	Neuropsychiatric and respiratory diseases	Eye diseases	MSK, respiratory, and immune diseases	Unspecific group	Vascular diseases	Cardiometabolic diseases	Respiratory diseases	Neuropsychiatric diseases	Eye and MSK diseases	Unspecific group
Number (n)	159	272	313	309	460	1418	101	170	201	235	270	740	63	112	120	122	200	400
Age (mean)	73.3 (73.0-73.7)	82.3 (81.9-82.6)	83.2 (82.9-83.5)	88.2 (87.9-88.5)	73.9 (73.5-74.3)	71.8 (71.5-72.1)	82.0 (81.6-82.4)	85.5 (85.1-85.9)	80.8 (80.3-81.3)	84.6 (84.2-85)	75.5 (75.1-75.9)	74.0 (73.3-74.4)	84.0 (83.5-84.5)	84.0 (83.6-84.5)	79.8 (79.4-80.3)	87.4 (86.9-87.8)	81.8 (81.4-82.3)	76.8 (76.4-77.1)
Sex (female, %)	74.2 (66.9-80.3)	59.2 (53.3-64.9)	72.5 (37.3-77.2)	76.9 (71.9-81.2)	74.4 (70.2-78.1)	61.0 (58.5-63.5)	51.0 (41.4-60.6)	57.3 (49.8-64.5)	72.9 (66.4-78.6)	76.6 (70.8-81.6)	72.4 (66.8-77.4)	59.3 (55.7-62.8)	73.3 (61.3-81.7)	44.2 (35.4-53.5)	77.5 (69.2-84.0)	70.6 (62.0-77.9)	77.6 (71.3-82.8)	58.7 (53.8-63.4)
Education																		
Elementary (%)	16.9 (11.9-23.5)	25.1 (20.3-30.6)	21.8 (17.5-26.7)	34.8 (29.6-40.5)	19.3 (16.0-23.2)	14.4 (12.7-16.3)	19.1 (12.6-27.8)	19.6 (14.3-26.3)	15.5 (11.2-21.2)	20.5 (15.8-26.1)	8.6 (5.8-12.5)	10.4 (8.4-12.8)	21.6 (13.2-33.2)	11.7 (6.9-18.9)	8.9 (5.0-15.3)	20.0 (13.9-27.9)	8.4 (5.3-13.1)	5.4 (3.6-8.0)
Secondary (%)	47.6 (33.9-55.3)	56.1 (50.1-61.9)	56.5 (50.9-61.9)	47.4 (41.8-53.2)	49.5 (45.0-54.1)	50.1 (47.5-52.7)	51.4 (41.8-61.0)	50.9 (43.4-58.3)	50.3 (43.5-57.2)	53.2 (46.8-59.5)	49.4 (43.5-55.3)	48.0 (44.4-51.6)	52.0 (40.0-63.9)	47.2 (38.2-56.4)	40.9 (32.5-49.8)	52.7 (43.9-61.3)	49.0 (42.2-55.9)	46.4 (41.5-51.3)
University (%)	35.5 (28.5-43.2)	18.8 (14.6-	21.8 (27.6-26.7)	17.7 (13.8-22.5)	31.1 (27.1-	35.5 (33.0-38.0)	29.5 (21.5-	29.5 (23.1-	34.1 (27.9-41.0)	26.3 (21.1-	42.0 (36.3-	41.6 (38.1-45.2)	26.4 (17.1-38.4)	41.1 (32.4-50.4)	50.2 (41.4-59.0)	27.3 (20.2-35.8)	42.6 (35.9-49.5)	48.3 (43.4-

		23.9)			35.5)		39.0)	36.8)		32.3)	48.0)							53.2)
Number of diseases (mean)	5.7 (5.6-5.8)	7.7 (7.6-7.8)	6.0 (6.0-6.1)	5.5 (5.5-5.6)	4.7 (4.6-4.7)	3.2 (3.2-3.3)	11.5 (11.4-11.7)	10.9 (10.7-11.0)	8.9 (8.8-9.1)	9.3 (9.1-9.4)	7.8 (7.7-7.9)	4.8 (4.7-4.9)	14.9 (14.6-15.1)	13.3 (13.1-13.5)	12.2 (12.0-12.5)	15.0 (14.7-15.2)	10.7 (10.6-10.9)	6.6 (5.5-6.7)
Number of drugs (mean)	6.2 (6.1-6.4)	7.7 (7.6-7.8)	5.1 (4.9-5.2)	6.1 (5.9-6.2)	5.3 (5.2-5.4)	2.9 (2.8-2.9)	8.3 (8.1-8.5)	8.8 (8.6-9.0)	7.8 (7.6-8.0)	6.6 (6.4-6.8)	6.3 (6.2-6.5)	3.8 (3.7-3.9)	9.6 (9.3-9.9)	8.7 (8.4-9.0)	8.8 (8.5-9.0)	9.4 (9.2-9.7)	6.4 (6.2-6.7)	4.5 (4.3-4.6)
Walking speed <0.8 m/s (%)	35.7 (28.5-43.5)	63.1 (57.1-68.7)	51.0 (45.3-56.5)	83.8 (12.1-21.3)	31.2 (27.1-35.6)	16.0 (14.2-18.0)	51.2 (51.5-60.8)	67.7 (60.2-74.3)	54.4 (47.5-61.2)	59.5 (53.1-65.7)	30.3 (25.2-36.1)	15.5 (13.1-18.3)	63.9 (51.4-74.7)	55.9 (46.6-64.7)	39.3 (31.1-48.3)	79.6 (71.6-85.8)	33.9 (27.7-40.8)	15.0 (11.9-18.9)
Disability score ≥1 (%)	32.5 (17.9-31.6)	49.9 (30.1-41.8)	38.4 (24.0-34.2)	86.1 (77.9-86.6)	18.4 (9.0-15.0)	8.8 (3.5-5.7)	23.0 (7.3-20.4)	24.3 (11.5-22.9)	22.7 (11.3-21.8)	17.3 (7.1-15.1)	7.3 (1.4-5.7)	2.8 (0.5-2.0)	9.7 (2.4-15.3)	5.7 (0.3-5.8)	4.7 (0.3-5.4)	11.1 (2.4-10.7)	2.2 (0.0-2.1)	1.1 (0.0-1.0)
MMSE (mean)	27.7 (27.5-27.9)	27.6 (27.5-27.7)	27.7 (27.6-27.8)	16.6 (16.3-17.0)	28.6 (28.5-28.7)	28.8 (28.7-28.8)	26.7 (26.5-26.9)	24.3 (24.0-24.7)	24.7 (24.4-25.1)	26.4 (26.1-26.6)	28.2 (28.1-28.3)	28.2 (28.1-28.3)	26.5 (26.2-26.8)	26.3 (26.0-26.7)	27.2 (27.0-27.5)	22.1 (21.6-22.5)	27.9 (27.7-28.1)	28.1 (27.9-28.3)
Hypertension (%)	65.4 (57.7-72.3)	73.4 (67.9-78.3)	80.6 (75.8-84.6)	42.4 (37.0-48.0)	57.8 (53.3-62.3)	84.3 (82.3-86.1)	93.7 (87.2-97.0)	91.1 (85.8-94.5)	75.2 (68.8-80.6)	90.3 (85.9-93.5)	80.2 (75.-84.5)	90.2 (87.8-92.1)	94.1 (85.4-97.7)	96.3 (81.0-98.6)	81.5 (73.6-87.5)	96.6 (91.8-98.7)	89.7 (84.7-93.2)	89.7 (86.3-92.3)
Diabetes (%)	6.4 (3.5-11.3)	30.6 (25.4-36.3)	11.5 (8.4-15.5)	4.9 (3.0-7.9)	6.7 (4.8-9.4)	8.2 (6.9-9.8)	31.1 (22.9-40.7)	24.6 (18.7-31.6)	9.4 (6.1-14.3)	12.3 (8.7-17.1)	9.6 (6.6-13.7)	13.4 (11.1-16.0)	24.6 (15.6-36.4)	46.7 (37.7-55.9)	12.7 (7.8-19.8)	20.2 (14.0-28.1)	10.1 (6.6-15.1)	12.1 (9.3-15.7)
Dyslipidemia (%)	48.1 (40.5-55.9)	40.9 (35.2-46.8)	44.8 (39.4-50.3)	12.9 (9.6-17.1)	42.0 (37.5-46.5)	64.1 (61.5-66.5)	59.7 (50.0-68.8)	59.4 (51.8-66.5)	58.7 (51.8-65.3)	56.8 (50.4-62.9)	57.9 (52.0-63.7)	77.8 (74.7-80.6)	69.9 (57.7-79.8)	76.8 (68.1-83.6)	66.8 (58.0-74.6)	60.9 (52.1-69.1)	78.0 (71.8-83.2)	84.8 (81.0-88.0)

Obesity (%)	8.3 (5.0-13.7)	16.0 (12.2-20.9)	7.3 (4.9-10.8)	0.7 (0.2-2.4)	17.8 (14.6-21.6)	15.4 (13.7-17.4)	16.6 (10.6-25.1)	16.4 (11.6-22.8)	13.4 (9.4-18.8)	13.3 (9.6-18.3)	24.2 (19.5-29.7)	24.7 (21.7-27.9)	23.6 (14.8-35.4)	29.7 (22.0-38.7)	26.1 (19.1-34.6)	26.4 (19.4-34.9)	18.9 (14.1-24.9)	27.2 (23.0-31.7)

Supplementary table 2. Additional clinical and drug-related* parameters used in SNAC-K for specific chronic conditions.

*The ATC codes corresponding to each drug are shown in brackets. Only those drugs that can be unequivocally linked to chronic conditions were considered. That is, drugs with more than one indication were excluded from the list. The selection of ATC codes was based on a literature review and the clinical judgement of physicians.

NOTE: The criteria presented in this table were used in addition to the diagnoses assigned in SNAC-K. For example, use of dopaminergic agents was considered to indicate presence of Parkinson syndrome, even in the absence of other diagnostic information.

Condition	Clinical and drug-related parameters
**Anemia**	Hemoglobin <13 g/dl in men and <12 g/dl in women<br>Use of iron preparations (B03A) or other antianemic preparations (B03XA)
**Asthma**	Use of leukotriene receptor antagonists (R03DC) or antiallergic agents, excl. corticosteroids (R03BC)
**Atrial fibrillation**	Discrete P wave undetectable and irregular ventricular rate (12-lead electrocardiogram)
**Autoimmune diseases**	Use of antipsoriatics (D05)
**Bradycardias and conduction diseases**	Presence of a cardiac pacemaker (12-lead electrocardiogram)
**Chronic infectious diseases**	Use of drugs for treatment of tuberculosis excluding cycloserine, rifampicine, rifamicyne and hydrazides (J04A excl. J04AB01, J04AB02, J04AB03 and J04AC)
**Chronic kidney diseases**	Glomerular filtration rate <60 ml/min/1.73m2 (assessed using the CKD-EPI equation)
**Chronic pancreas, biliary tract and gallbladder diseases**	Use of multienzymes (lipase, protease etc.) (A09AA02)
**Colitis and related diseases**	Use of drugs for constipation (A06A)
**COPD, Emphysema, Chronic Bronchitis**	Use of anticholinergics (R03BB)
**Dementia**	Diagnostic and Statistical Manual of Mental Disorders, Third Edition, Revised (assessed by two different physicians, and a third one in case of disagreement)

	Use of anticholinesterases (N06DA) or memantine (N06DX01)
**Diabetes**	Glycated hemoglobin (A1C) ≥6.5%<br>Use of antidiabetics (A10)
**Esophagus, stomach and duodenum diseases**	Use of other drugs for peptic ulcer and gastro-oesophageal reflux disease (A02BX)
**Glaucoma**	Use of beta blocking agents (S01ED)
**Hearing impairment**	Unable to hear the interviewer's voice at a normal volume (assessed by a nurse)
**Hypercholesterolemia**	Serum total cholesterol ≥6.22 mmol/L
**Hypertension**	Blood pressure ≥140/90 mmHg
**Inflammatory arthropathies**	Use of gold preparations (M01CB)
**Inflammatory bowel diseases**	Use of intestinal antiinflammatory agents (A07E)
**Ischemic heart disease**	Use of organic nitrates (C01DA) or ranolazine (C01EB18)
**Migraine and facial pain syndromes**	Use of antimigraine preparations (N02C)
**Obesity**	Body Mass Index ≥30 kg/m^2
**Osteoporosis**	Use of bisphosphonates (M05BA), bisphosphonate combinations (M05BB), strontium ranelate (M05BX03) or strontium ranelate and colecalciferol (M05BX53)
**Other psychiatric and behavioral diseases**	Use of drugs for alcohol dependence (N07BB)
**Parkinson and parkinsonism**	Use of dopa and dopa derivatives (N04BA), dopamine agonists (N04BC), or other dopaminergic agents (N04BX)
**Peripheral vascular disease**	Use of cilostazol (B01AC23)
**Prostate diseases**	Use of drugs for benign prostatic hypertrophy excluding testosterone-5-alpha reductase inhibitors (G04C excl. G04CB)
**Thyroid diseases**	Use of thyroid hormones (H03AA) or antithyroid preparations (H03B)
**Visual impairment**	Unable to see the physician at a close distance with or without aid (assessed by a nurse)

1. **Supplementary table 3. Descriptors of ICD-10 codes included and excluded in each chronic disease category.**

   NOTE: When all sub-codes within a given ICD-10 code were classified as chronic, the highest possible level of aggregation of the hierarchy was included in the list (e.g. three-digit code for asthma (J45), one-digit code for malignant neoplasms (C), etc.

**ALLERGY**	
**Included ICD-10 codes and labels**	
J301	Allergic rhinitis due to pollen
J302	Other seasonal allergic rhinitis
J303	Other allergic rhinitis
J304	Allergic rhinitis, unspecified
J450	Predominantly allergic asthma
K522	Allergic and dietetic gastroenteritis and colitis
L20	Atopic dermatitis
L23	Allergic contact dermatitis
L500	Allergic urticaria
Z516	Desensitization to allergens
**ANEMIA**	
**Included ICD-10 codes and labels**	
D50	Iron deficiency anaemia
D51	Vitamin B12 deficiency anaemia
D52	Folate deficiency anaemia
D53	Other nutritional anaemias
D55	Anaemia due to enzyme disorders
D56	Thalassaemia
D57	Sickle-cell disorders
D58	Other hereditary haemolytic anaemias
D59	Acquired haemolytic anaemia
D60	Acquired pure red cell aplasia [erythroblastopenia]
D61	Other aplastic anaemias
D63	Anaemia in chronic diseases classified elsewhere
D64	Other anaemias
**Excluded ICD-10 codes and labels**	
D563	Thalassaemia trait
D590	Drug-induced autoimmune haemolytic anaemia
D592	Drug-induced nonautoimmune haemolytic anaemia
D593	Haemolytic-uraemic syndrome
D596	Haemoglobinuria due to haemolysis from other external causes
D601	Transient acquired pure red cell aplasia
D611	Drug-induced aplastic anaemia
D612	Aplastic anaemia due to other external agents
D642	Secondary sideroblastic anaemia due to drugs and toxins

**ASTHMA**	
**Included ICD-10 codes and labels**	
J45	Asthma
**ATRIAL FIBRILLATION**	
**Included ICD-10 codes and labels**	
I48	Atrial fibrillation and flutter
**AUTOIMMUNE DISEASES**	
**Included ICD-10 codes and labels**	
I731	Thromboangiitis obliterans [Buerger]
L10	Pemphigus
L12	Pemphigoid
L40	Psoriasis
L41	Parapsoriasis
L93	Lupus erythematosus
L94	Other localized connective tissue disorders
L95	Vasculitis limited to skin, not elsewhere classified
M30	Polyarteritis nodosa and related conditions
M31	Other necrotizing vasculopathies
M32	Systemic lupus erythematosus
M33	Dermatopolymyositis
M34	Systemic sclerosis
M35	Other systemic involvement of connective tissue
M36	Systemic disorders of connective tissue in diseases classified elsewhere
**Excluded ICD-10 codes and labels**	
L105	Drug-induced pemphigus
M320	Drug-induced systemic lupus erythematosus
M342	Systemic sclerosis induced by drugs and chemicals
M357	Hypermobility syndrome
M358	Other specified systemic involvement of connective tissue
M359	Systemic involvement of connective tissue, unspecified
M360	Dermato(poly)myositis in neoplastic disease
M361	Arthropathy in neoplastic disease
M362	Haemophilic arthropathy
M363	Arthropathy in other blood disorders
**BLINDNESS, VISUAL IMPAIRMENT**	
**Included ICD-10 codes and labels**	
H54	Visual impairment including blindness (binocular or monocular)
Z442	Fitting and adjustment of artificial eye
Z970	Presence of artificial eye
**Excluded ICD-10 codes and labels**	
H543	Mild or no visual impairment, binocular
**BLOOD AND BLOOD FORMING ORGAN DISEASES**	
**Included ICD-10 codes and labels**	

D66	Hereditary factor VIII deficiency
D67	Hereditary factor IX deficiency
D68	Other coagulation defects
D69	Purpura and other haemorrhagic conditions
D71	Functional disorders of polymorphonuclear neutrophils
D720	Genetic anomalies of leukocytes
D730	Hyposplenism
D731	Hypersplenism
D732	Chronic congestive splenomegaly
D74	Methaemoglobinaemia
D750	Familial erythrocytosis
D761	Haemophagocytic lymphohistiocytosis
D763	Other histiocytosis syndromes
D77	Other disorders of blood and blood-forming organs in diseases classified elsewhere
D80	Immunodeficiency with predominantly antibody defects
D81	Combined immunodeficiencies
D82	Immunodeficiency associated with other major defects
D83	Common variable immunodeficiency
D84	Other immunodeficiencies
D86	Sarcoidosis
D89	Other disorders involving the immune mechanism, not elsewhere classified
**Excluded ICD-10 codes and labels**	
D683	Haemorrhagic disorder due to circulating anticoagulants
D684	Acquired coagulation factor deficiency
D695	Secondary thrombocytopenia
D748	Other methaemoglobinaemias
D807	Transient hypogammaglobulinaemia of infancy
D891	Cryoglobulinaemia
D893	Immune reconstitution syndrome
**BRADYCARDIAS AND CONDUCTION DISEASES**	
**Included ICD-10 codes and labels**	
I441	Atrioventricular block, second degree
I442	Atrioventricular block, complete
I443	Other and unspecified atrioventricular block
I453	Trifascicular block
I455	Other specified heart block
Z950	Presence of cardiac pacemaker
**CARDIAC VALVE DISEASES**	
**Included ICD-10 codes and labels**	
I05	Rheumatic mitral valve diseases
I06	Rheumatic aortic valve diseases
I07	Rheumatic tricuspid valve diseases
I08	Multiple valve diseases

I091	Rheumatic diseases of endocardium, valve unspecified
I098	Other specified rheumatic heart diseases
I34	Nonrheumatic mitral valve disorders
I35	Nonrheumatic aortic valve disorders
I36	Nonrheumatic tricuspid valve disorders
I37	Pulmonary valve disorders
I38	Endocarditis, valve unspecified
I390	Mitral valve disorders in diseases classified elsewhere
I391	Aortic valve disorders in diseases classified elsewhere
I392	Tricuspid valve disorders in diseases classified elsewhere
I393	Pulmonary valve disorders in diseases classified elsewhere
I394	Multiple valve disorders in diseases classified elsewhere
Q22	Congenital malformations of pulmonary and tricuspid valves
Q23	Congenital malformations of aortic and mitral valves
Z952	Presence of prosthetic heart valve
Z953	Presence of xenogenic heart valve
Z954	Presence of other heart-valve replacement
**CATARACT AND OTHER LENS DISEASES**	
**Included ICD-10 codes and labels**	
H25	Senile cataract
H26	Other cataract
H27	Other disorders of lens
H28	Cataract and other disorders of lens in diseases classified elsewhere
Q12	Congenital lens malformations
Z961	Presence of intraocular lens
**CEREBROVASCULAR DISEASE**	
**Included ICD-10 codes and labels**	
G45	Transient cerebral ischaemic attacks and related syndromes
G46	Vascular syndromes of brain in cerebrovascular diseases
I60	Subarachnoid haemorrhage
I61	Intracerebral haemorrhage
I62	Other nontraumatic intracranial haemorrhage
I63	Cerebral infarction
I64	Stroke, not specified as haemorrhage or infarction
I67	Other cerebrovascular diseases
I69	Sequelae of cerebrovascular disease
**CHROMOSOMAL ABNORMALITIES**	
**Included ICD-10 codes and labels**	
Q90	Down syndrome
Q91	Edwards syndrome and Patau syndrome
Q92	Other trisomies and partial trisomies of the autosomes, not elsewhere classified
Q93	Monosomies and deletions from the autosomes, not elsewhere classified
Q95	Balanced rearrangements and structural markers, not elsewhere classified

Q96	Turner syndrome
Q97	Other sex chromosome abnormalities, female phenotype, not elsewhere classified
Q98	Other sex chromosome abnormalities, male phenotype, not elsewhere classified
Q99	Other chromosome abnormalities, not elsewhere classified
**CHRONIC INFECTIOUS DISEASES**	
**Included ICD-10 codes and labels**	
A15	Respiratory tuberculosis, bacteriologically and histologically confirmed
A16	Respiratory tuberculosis, not confirmed bacteriologically or histologically
A17	Tuberculosis of nervous system
A18	Tuberculosis of other organs
A19	Miliary tuberculosis
A30	Leprosy [Hansen disease]
A31	Infection due to other mycobacteria
A50	Congenital syphilis
A52	Late syphilis
A53	Other and unspecified syphilis
A65	Nonvenereal syphilis
A66	Yaws
A67	Pinta [carate]
A692	Lyme disease
A81	Atypical virus infections of central nervous system
B20	Human immunodeficiency virus [HIV] disease resulting in infectious and parasitic diseases
B21	Human immunodeficiency virus [HIV] disease resulting in malignant neoplasms
B22	Human immunodeficiency virus [HIV] disease resulting in other specified diseases
B23	Human immunodeficiency virus [HIV] disease resulting in other conditions
B24	Unspecified human immunodeficiency virus [HIV] disease
B381	Chronic pulmonary coccidioidomycosis
B391	Chronic pulmonary histoplasmosis capsulati
B401	Chronic pulmonary blastomycosis
B572	Chagas disease (chronic) with heart involvement
B573	Chagas disease (chronic) with digestive system involvement
B574	Chagas disease (chronic) with nervous system involvement
B575	Chagas disease (chronic) with other organ involvement
B65	Schistosomiasis [bilharziasis]
B92	Sequelae of leprosy
B94	Sequelae of other and unspecified infectious and parasitic diseases
J65	Pneumoconiosis associated with tuberculosis
M863	Chronic multifocal osteomyelitis
M864	Chronic osteomyelitis with draining sinus
M865	Other chronic haematogenous osteomyelitis
M866	Other chronic osteomyelitis
**CHRONIC KIDNEY DISEASES**	
**Included ICD-10 codes and labels**	

I120	Hypertensive renal disease with renal failure
I130	Hypertensive heart and renal disease with (congestive) heart failure
I131	Hypertensive heart and renal disease with renal failure
I132	Hypertensive heart and renal disease with both (congestive) heart failure and renal failure
I139	Hypertensive heart and renal disease, unspecified
N01	Rapidly progressive nephritic syndrome
N03	Chronic nephritic syndrome
N04	Nephrotic syndrome
N05	Unspecified nephritic syndrome
N07	Hereditary nephropathy, not elsewhere classified
N08	Glomerular disorders in diseases classified elsewhere
N11	Chronic tubulo-interstitial nephritis
N183	Chronic kidney disease, stage 3
N184	Chronic kidney disease, stage 4
N185	Chronic kidney disease, stage 5
N189	Chronic kidney disease, unspecified
Q60	Renal agenesis and other reduction defects of kidney
Q611	Polycystic kidney, autosomal recessive
Q612	Polycystic kidney, autosomal dominant
Q613	Polycystic kidney, unspecified
Q614	Renal dysplasia
Q615	Medullary cystic kidney
Q618	Other cystic kidney diseases
Q619	Cystic kidney disease, unspecified
Z905	Acquired absence of kidney
Z940	Kidney transplant status
**CHRONIC LIVER DISEASES**	
**Included ICD-10 codes and labels**	
B18	Chronic viral hepatitis
K70	Alcoholic liver disease
K713	Toxic liver disease with chronic persistent hepatitis
K714	Toxic liver disease with chronic lobular hepatitis
K715	Toxic liver disease with chronic active hepatitis
K717	Toxic liver disease with fibrosis and cirrhosis of liver
K721	Chronic hepatic failure
K73	Chronic hepatitis, not elsewhere classified
K74	Fibrosis and cirrhosis of liver
K753	Granulomatous hepatitis, not elsewhere classified
K754	Autoimmune hepatitis
K758	Other specified inflammatory liver diseases
K761	Chronic passive congestion of liver
K766	Portal hypertension
K767	Hepatorenal syndrome

K778	Liver disorders in other diseases classified elsewhere
Q446	Cystic disease of liver
Z944	Liver transplant status
**Excluded ICD-10 codes and labels**	
K700	Alcoholic fatty liver
K701	Alcoholic hepatitis
**CHRONIC PANCREAS, BILIARY TRACT AND GALLBLADDER DISEASES**	
**Included ICD-10 codes and labels**	
K800	Calculus of gallbladder with acute cholecystitis
K801	Calculus of gallbladder with other cholecystitis
K802	Calculus of gallbladder without cholecystitis
K808	Other cholelithiasis
K811	Chronic cholecystitis
K86	Other diseases of pancreas
Q440	Agenesis, aplasia and hypoplasia of gallbladder
Q441	Other congenital malformations of gallbladder
Q442	Atresia of bile ducts
Q443	Congenital stenosis and stricture of bile ducts
Q444	Choledochal cyst
Q445	Other congenital malformations of bile ducts
Q450	Agenesis, aplasia and hypoplasia of pancreas
**Excluded ICD-10 codes and labels**	
K862	Cyst of pancreas
K863	Pseudocyst of pancreas
K869	Disease of pancreas, unspecified
**CHRONIC ULCER OF THE SKIN**	
**Included ICD-10 codes and labels**	
I830	Varicose veins of lower extremities with ulcer
I832	Varicose veins of lower extremities with both ulcer and inflammation
L89	Decubitus ulcer and pressure area
L97	Ulcer of lower limb, not elsewhere classified
L984	Chronic ulcer of skin, not elsewhere classified
**COLITIS AND RELATED DISEASES**	
**Included ICD-10 codes and labels**	
K520	Gastroenteritis and colitis due to radiation
K528	Other specified noninfective gastroenteritis and colitis
K551	Chronic vascular disorders of intestine
K552	Angiodysplasia of colon
K572	Diverticular disease of large intestine with perforation and abscess
K573	Diverticular disease of large intestine without perforation or abscess
K574	Diverticular disease of both small and large intestine with perforation and abscess
K575	Diverticular disease of both small and large intestine without perforation or abscess
K578	Diverticular disease of intestine, part unspecified, with perforation and abscess

K579	Diverticular disease of intestine, part unspecified, without perforation or abscess
K58	Irritable bowel syndrome
K590	Constipation
K592	Neurogenic bowel, not elsewhere classified
K62	Other diseases of anus and rectum
K634	Enteroptosis
K64	Haemorrhoids and perianal venous thrombosis
**Excluded ICD-10 codes and labels**	
K620	Anal polyp
K621	Rectal polyp
K625	Haemorrhage of anus and rectum
K626	Ulcer of anus and rectum
K645	Perianal venous thrombosis
**COPD, EMPHYSEMA, CHRONIC BRONCHITIS**	
**Included ICD-10 codes and labels**	
J41	Simple and mucopurulent chronic bronchitis
J42	Unspecified chronic bronchitis
J43	Emphysema
J44	Other chronic obstructive pulmonary disease
J47	Bronchiectasis
**DEAFNESS, HEARING IMPAIRMENT**	
**Included ICD-10 codes and labels**	
H80	Otosclerosis
H90	Conductive and sensorineural hearing loss
H911	Presbycusis
H913	Deaf mutism, not elsewhere classified
H919	Hearing loss, unspecified
Q16	Congenital malformations of ear causing impairment of hearing
Z453	Adjustment and management of implanted hearing device
Z461	Fitting and adjustment of hearing aid
Z962	Presence of otological and audiological implants
Z974	Presence of external hearing-aid
**DEMENTIA**	
**Included ICD-10 codes and labels**	
F00	Dementia in Alzheimer disease
F01	Vascular dementia
F02	Dementia in other diseases classified elsewhere
F03	Unspecified dementia
F051	Delirium superimposed on dementia
G30	Alzheimer disease
G31	Other degenerative diseases of nervous system, not elsewhere classified
**DEPRESSION AND MOOD DISEASES**	
**Included ICD-10 codes and labels**	

F30	Manic episode
F31	Bipolar affective disorder
F32	Depressive episode
F33	Recurrent depressive disorder
F34	Persistent mood [affective] disorders
F38	Other mood [affective] disorders
F39	Unspecified mood [affective] disorder
F412	Mixed anxiety and depressive disorder
**DIABETES**	
**Included ICD-10 codes and labels**	
E10	Insulin-dependent diabetes mellitus
E11	Non-insulin-dependent diabetes mellitus
E13	Other specified diabetes mellitus
E14	Unspecified diabetes mellitus
E891	Postprocedural hypoinsulinaemia
**DORSOPATHIES**	
**Included ICD-10 codes and labels**	
M40	Kyphosis and lordosis
M41	Scoliosis
M42	Spinal osteochondrosis
M43	Other deforming dorsopathies
M47	Spondylosis
M48	Other spondylopathies
M49	Spondylopathies in diseases classified elsewhere
M50	Cervical disc disorders
M51	Other intervertebral disc disorders
M53	Other dorsopathies, not elsewhere classified
Q675	Congenital deformity of spine
Q761	Klippel-Feil syndrome
Q764	Other congenital malformations of spine, not associated with scoliosis
**DYSLIPIDEMIA**	
**Included ICD-10 codes and labels**	
E78	Disorders of lipoprotein metabolism and other lipidaemias
**EAR, NOSE, THROAT DISEASES**	
**Included ICD-10 codes and labels**	
H604	Cholesteatoma of external ear
H661	Chronic tubotympanic suppurative otitis media
H662	Chronic atticoantral suppurative otitis media
H663	Other chronic suppurative otitis media
H701	Chronic mastoiditis
H71	Cholesteatoma of middle ear
H731	Chronic myringitis
H741	Adhesive middle ear disease

H810	MÚniÞre disease
H831	Labyrinthine fistula
H832	Labyrinthine dysfunction
H95	Postprocedural disorders of ear and mastoid process, not elsewhere classified
J300	Vasomotor rhinitis
J31	Chronic rhinitis, nasopharyngitis and pharyngitis
J32	Chronic sinusitis
J33	Nasal polyp
J341	Cyst and mucocele of nose and nasal sinus
J342	Deviated nasal septum
J343	Hypertrophy of nasal turbinates
J35	Chronic diseases of tonsils and adenoids
J37	Chronic laryngitis and laryngotracheitis
J380	Paralysis of vocal cords and larynx
J386	Stenosis of larynx
K051	Chronic gingivitis
K053	Chronic periodontitis
K07	Dentofacial anomalies [including malocclusion]
K110	Atrophy of salivary gland
K117	Disturbances of salivary secretion
Q30	Congenital malformations of nose
Q31	Congenital malformations of larynx
Q32	Congenital malformations of trachea and bronchus
Q35	Cleft palate
Q36	Cleft lip
Q37	Cleft palate with cleft lip
Q38	Other congenital malformations of tongue, mouth and pharynx
**EPILEPSY**	
**Included ICD-10 codes and labels**	
G40	Epilepsy
**Excluded ICD-10 codes and labels**	
G405	Special epileptic syndromes
**ESOPHAGUS, STOMACH AND DUODENUM DISEASES**	
**Included ICD-10 codes and labels**	
I85	Oesophageal varices
I864	Gastric varices
I982	Oesophageal varices without bleeding in diseases classified elsewhere
I983	Oesophageal varices with bleeding in diseases classified elsewhere
K21	Gastro-oesophageal reflux disease
K220	Achalasia of cardia
K222	Oesophageal obstruction
K224	Dyskinesia of oesophagus
K225	Diverticulum of oesophagus, acquired

K227	Barrett oesophagus
K230	Tuberculous oesophagitis
K231	Megaoesophagus in Chagas disease
K254	Gastric ulcer: Chronic or unspecified with haemorrhage
K255	Gastric ulcer: Chronic or unspecified with perforation
K256	Gastric ulcer: Chronic or unspecified with both haemorrhage and perforation
K257	Gastric ulcer: Chronic without haemorrhage or perforation
K264	Duodenal ulcer: Chronic or unspecified with haemorrhage
K265	Duodenal ulcer: Chronic or unspecified with perforation
K266	Duodenal ulcer: Chronic or unspecified with both haemorrhage and perforation
K267	Duodenal ulcer: Chronic without haemorrhage or perforation
K274	Peptic ulcer, site unspecified: Chronic or unspecified with haemorrhage
K275	Peptic ulcer, site unspecified: Chronic or unspecified with perforation
K276	Peptic ulcer, site unspecified: Chronic or unspecified with both haemorrhage and perforation
K277	Peptic ulcer, site unspecified: Chronic without haemorrhage or perforation
K284	Gastrojejunal ulcer: Chronic or unspecified with haemorrhage
K285	Gastrojejunal ulcer: Chronic or unspecified with perforation
K286	Gastrojejunal ulcer: Chronic or unspecified with both haemorrhage and perforation
K287	Gastrojejunal ulcer: Chronic without haemorrhage or perforation
K293	Chronic superficial gastritis
K294	Chronic atrophic gastritis
K295	Chronic gastritis, unspecified
K296	Other gastritis
K297	Gastritis, unspecified
K298	Duodenitis
K299	Gastroduodenitis, unspecified
K311	Adult hypertrophic pyloric stenosis
K312	Hourglass stricture and stenosis of stomach
K313	Pylorospasm, not elsewhere classified
K314	Gastric diverticulum
K315	Obstruction of duodenum
Q39	Congenital malformations of oesophagus
Q40	Other congenital malformations of upper alimentary tract
Z903	Acquired absence of part of stomach
**GLAUCOMA**	
**Included ICD-10 codes and labels**	
H401	Primary open-angle glaucoma
H402	Primary angle-closure glaucoma
H403	Glaucoma secondary to eye trauma
H404	Glaucoma secondary to eye inflammation
H405	Glaucoma secondary to other eye disorders
H406	Glaucoma secondary to drugs
H408	Other glaucoma

H409	Glaucoma, unspecified
**HEART FAILURE**	
**Included ICD-10 codes and labels**	
I110	Hypertensive heart disease with (congestive) heart failure
I130	Hypertensive heart and renal disease with (congestive) heart failure
I132	Hypertensive heart and renal disease with both (congestive) heart failure and renal failure
I27	Other pulmonary heart diseases
I280	Arteriovenous fistula of pulmonary vessels
I42	Cardiomyopathy
I43	Cardiomyopathy in diseases classified elsewhere
I50	Heart failure
I515	Myocardial degeneration
I517	Cardiomegaly
I528	Other heart disorders in other diseases classified elsewhere
Z941	Heart transplant status
Z943	Heart and lungs transplant status
**HEMATOLOGICAL NEOPLASMS**	
**Included ICD-10 codes and labels**	
C81	Hodgkin lymphoma
C82	Follicular lymphoma
C83	Non-follicular lymphoma
C84	Mature T/NK-cell lymphomas
C85	Other and unspecified types of non-Hodgkin lymphoma
C86	Other specified types of T/NK-cell lymphoma
C88	Malignant immunoproliferative diseases
C90	Multiple myeloma and malignant plasma cell neoplasms
C91	Lymphoid leukaemia
C92	Myeloid leukaemia
C93	Monocytic leukaemia
C94	Other leukaemias of specified cell type
C95	Leukaemia of unspecified cell type
C96	Other and unspecified malignant neoplasms of lymphoid, haematopoietic and related tissue
**HYPERTENSION**	
**Included ICD-10 codes and labels**	
I10	Essential (primary) hypertension
I11	Hypertensive heart disease
I12	Hypertensive renal disease
I13	Hypertensive heart and renal disease
I15	Secondary hypertension
**INFLAMMATORY ARTHROPATHIES**	
**Included ICD-10 codes and labels**	
M023	Reiter disease
M05	Seropositive rheumatoid arthritis

M06	Other rheumatoid arthritis
M07	Psoriatic and enteropathic arthropathies
M08	Juvenile arthritis
M09	Juvenile arthritis in diseases classified elsewhere
M10	Gout
M11	Other crystal arthropathies
M12	Other specific arthropathies
M13	Other arthritis
M14	Arthropathies in other diseases classified elsewhere
M45	Ankylosing spondylitis
M460	Spinal enthesopathy
M461	Sacroiliitis, not elsewhere classified
M468	Other specified inflammatory spondylopathies
M469	Inflammatory spondylopathy, unspecified
**INFLAMMATORY BOWEL DISEASES**	
**Included ICD-10 codes and labels**	
K50	Crohn disease [regional enteritis]
K51	Ulcerative colitis
**ISCHEMIC HEART DISEASE**	
**Included ICD-10 codes and labels**	
I20	Angina pectoris
I21	Acute myocardial infarction
I22	Subsequent myocardial infarction
I24	Other acute ischaemic heart diseases
I25	Chronic ischaemic heart disease
Z951	Presence of aortocoronary bypass graft
Z955	Presence of coronary angioplasty implant and graft
**MIGRAINE AND FACIAL PAIN SYNDROMES**	
**Included ICD-10 codes and labels**	
G43	Migraine
G440	Cluster headache syndrome
G441	Vascular headache, not elsewhere classified
G442	Tension-type headache
G443	Chronic post-traumatic headache
G448	Other specified headache syndromes
G50	Disorders of trigeminal nerve
**MULTIPLE SCLEROSIS**	
**Included ICD-10 codes and labels**	
G35	Multiple sclerosis
**NEUROTIC, STRESS-RELATED AND SOMATOFORM DISEASES**	
**Included ICD-10 codes and labels**	
F40	Phobic anxiety disorders
F41	Other anxiety disorders

F42	Obsessive-compulsive disorder
F43	Reaction to severe stress, and adjustment disorders
F44	Dissociative [conversion] disorders
F45	Somatoform disorders
F48	Other neurotic disorders
**Excluded ICD-10 codes and labels**	
F430	Acute stress reaction
F432	Adjustment disorders
**OBESITY**	
**Included ICD-10 codes and labels**	
E66	Obesity
**OSTEOARTHRITIS AND OTHER DEGENERATIVE JOINT DISEASES**	
**Included ICD-10 codes and labels**	
M15	Polyarthrosis
M16	Coxarthrosis [arthrosis of hip]
M17	Gonarthrosis [arthrosis of knee]
M18	Arthrosis of first carpometacarpal joint
M19	Other arthrosis
M362	Haemophilic arthropathy
M363	Arthropathy in other blood disorders
**OSTEOPOROSIS**	
**Included ICD-10 codes and labels**	
M80	Osteoporosis with pathological fracture
M81	Osteoporosis without pathological fracture
M82	Osteoporosis in diseases classified elsewhere
**OTHER CARDIOVASCULAR DISEASES**	
**Included ICD-10 codes and labels**	
I09	Other rheumatic heart diseases
I281	Aneurysm of pulmonary artery
I310	Chronic adhesive pericarditis
I311	Chronic constrictive pericarditis
I456	Pre-excitation syndrome
I495	Sick sinus syndrome
I498	Other specified cardiac arrhythmias
I70	Atherosclerosis
I71	Aortic aneurysm and dissection
I72	Other aneurysm and dissection
I790	Aneurysm of aorta in diseases classified elsewhere
I791	Aortitis in diseases classified elsewhere
I950	Idiopathic hypotension
I951	Orthostatic hypotension
I958	Other hypotension
Q20	Congenital malformations of cardiac chambers and connections

Q21	Congenital malformations of cardiac septa
Q24	Other congenital malformations of heart
Q25	Congenital malformations of great arteries
Q26	Congenital malformations of great veins
Q27	Other congenital malformations of peripheral vascular system
Q28	Other congenital malformations of circulatory system
Z958	Presence of other cardiac and vascular implants and grafts
Z959	Presence of cardiac and vascular implant and graft, unspecified
**Excluded ICD-10 codes and labels**	
I091	Rheumatic diseases of endocardium, valve unspecified
I098	Other specified rheumatic heart diseases
I702	Atherosclerosis of arteries of extremities
**OTHER DIGESTIVE DISEASES**	
**Included ICD-10 codes and labels**	
K660	Peritoneal adhesions
K900	Coeliac disease
K901	Tropical sprue
K902	Blind loop syndrome, not elsewhere classified
K911	Postgastric surgery syndromes
K93	Disorders of other digestive organs in diseases classified elsewhere
Q41	Congenital absence, atresia and stenosis of small intestine
Q42	Congenital absence, atresia and stenosis of large intestine
Q43	Other congenital malformations of intestine
R15	Faecal incontinence
Z904	Acquired absence of other parts of digestive tract
Z980	Intestinal bypass and anastomosis status
**OTHER EYE DISEASES**	
**Included ICD-10 codes and labels**	
H022	Lagophthalmos
H023	Blepharochalasis
H024	Ptosis of eyelid
H025	Other disorders affecting eyelid function
H04	Disorders of lacrimal system
H05	Disorders of orbit
H104	Chronic conjunctivitis
H17	Corneal scars and opacities
H184	Corneal degeneration
H185	Hereditary corneal dystrophies
H186	Keratoconus
H187	Other corneal deformities
H188	Other specified disorders of cornea
H189	Disorder of cornea, unspecified
H193	Keratitis and keratoconjunctivitis in other diseases classified elsewhere

H198	Other disorders of sclera and cornea in diseases classified elsewhere
H201	Chronic iridocyclitis
H21	Other disorders of iris and ciliary body
H310	Chorioretinal scars
H311	Choroidal degeneration
H312	Hereditary choroidal dystrophy
H318	Other specified disorders of choroid
H319	Disorder of choroid, unspecified
H33	Retinal detachments and breaks
H352	Other proliferative retinopathy
H353	Degeneration of macula and posterior pole
H354	Peripheral retinal degeneration
H355	Hereditary retinal dystrophy
H357	Separation of retinal layers
H358	Other specified retinal disorders
H359	Retinal disorder, unspecified
H36	Retinal disorders in diseases classified elsewhere
H47	Other disorders of optic [2nd] nerve and visual pathways
H48	Disorders of optic [2nd] nerve and visual pathways in diseases classified elsewhere
H49	Paralytic strabismus
H51	Other disorders of binocular movement
Q10	Congenital malformations of eyelid, lacrimal apparatus and orbit
Q11	Anophthalmos, microphthalmos and macrophthalmos
Q13	Congenital malformations of anterior segment of eye
Q14	Congenital malformations of posterior segment of eye
Q15	Other congenital malformations of eye
Z947	Corneal transplant status
**Excluded ICD-10 codes and labels**	
H043	Acute and unspecified inflammation of lacrimal passages
H050	Acute inflammation of orbit
H470	Disorders of optic nerve, not elsewhere classified
H471	Papilloedema, unspecified
H481	Retrobulbar neuritis in diseases classified elsewhere
**OTHER GENITOURINARY DISEASES**	
**Included ICD-10 codes and labels**	
B901	Sequelae of genitourinary tuberculosis
N200	Calculus of kidney
N202	Calculus of kidney with calculus of ureter
N209	Urinary calculus, unspecified
N210	Calculus in bladder
N218	Other lower urinary tract calculus
N219	Calculus of lower urinary tract, unspecified
N22	Calculus of urinary tract in diseases classified elsewhere

N301	Interstitial cystitis (chronic)
N302	Other chronic cystitis
N303	Trigonitis
N304	Irradiation cystitis
N31	Neuromuscular dysfunction of bladder, not elsewhere classified
N320	Bladder-neck obstruction
N323	Diverticulum of bladder
N328	Other specified disorders of bladder
N329	Bladder disorder, unspecified
N33	Bladder disorders in diseases classified elsewhere
N35	Urethral stricture
N393	Stress incontinence
N394	Other specified urinary incontinence
N480	Leukoplakia of penis
N484	Impotence of organic origin
N489	Disorder of penis, unspecified
N701	Chronic salpingitis and oophoritis
N711	Chronic inflammatory disease of uterus
N731	Chronic parametritis and pelvic cellulitis
N734	Female chronic pelvic peritonitis
N736	Female pelvic peritoneal adhesions
N761	Subacute and chronic vaginitis
N763	Subacute and chronic vulvitis
N81	Female genital prolapse
N88	Other noninflammatory disorders of cervix uteri
N895	Stricture and atresia of vagina
N905	Atrophy of vulva
N952	Postmenopausal atrophic vaginitis
Q54	Hypospadias
Q620	Congenital hydronephrosis
Q621	Atresia and stenosis of ureter
Q622	Congenital megaloureter
Q623	Other obstructive defects of renal pelvis and ureter
Q624	Agenesis of ureter
Q627	Congenital vesico-uretero-renal reflux
Q628	Other congenital malformations of ureter
Q638	Other specified congenital malformations of kidney
Q639	Congenital malformation of kidney, unspecified
Q640	Epispadias
Q641	Exstrophy of urinary bladder
Q643	Other atresia and stenosis of urethra and bladder neck
Q644	Malformation of urachus
Q645	Congenital absence of bladder and urethra

Q646	Congenital diverticulum of bladder
Q647	Other congenital malformations of bladder and urethra
Q648	Other specified congenital malformations of urinary system
Q649	Congenital malformation of urinary system, unspecified
Z906	Acquired absence of other organs of urinary tract
Z907	Acquired absence of genital organ(s)
Z960	Presence of urogenital implants
**OTHER METABOLIC DISEASES**	
**Included ICD-10 codes and labels**	
E20	Hypoparathyroidism
E21	Hyperparathyroidism and other disorders of parathyroid gland
E22	Hyperfunction of pituitary gland
E23	Hypofunction and other disorders of pituitary gland
E24	Cushing syndrome
E25	Adrenogenital disorders
E26	Hyperaldosteronism
E27	Other disorders of adrenal gland
E28	Ovarian dysfunction
E29	Testicular dysfunction
E31	Polyglandular dysfunction
E34	Other endocrine disorders
E35	Disorders of endocrine glands in diseases classified elsewhere
E40	Kwashiorkor
E41	Nutritional marasmus
E42	Marasmic kwashiorkor
E43	Unspecified severe protein-energy malnutrition
E44	Protein-energy malnutrition of moderate and mild degree
E45	Retarded development following protein-energy malnutrition
E46	Unspecified protein-energy malnutrition
E64	Sequelae of malnutrition and other nutritional deficiencies
E70	Disorders of aromatic amino-acid metabolism
E71	Disorders of branched-chain amino-acid metabolism and fatty-acid metabolism
E72	Other disorders of amino-acid metabolism
E74	Other disorders of carbohydrate metabolism
E75	Disorders of sphingolipid metabolism and other lipid storage disorders
E76	Disorders of glycosaminoglycan metabolism
E77	Disorders of glycoprotein metabolism
E79	Disorders of purine and pyrimidine metabolism
E80	Disorders of porphyrin and bilirubin metabolism
E83	Disorders of mineral metabolism
E84	Cystic fibrosis
E85	Amyloidosis
E88	Other metabolic disorders

E89	Postprocedural endocrine and metabolic disorders, not elsewhere classified
K903	Pancreatic steatorrhoea
K904	Malabsorption due to intolerance, not elsewhere classified
K908	Other intestinal malabsorption
K909	Intestinal malabsorption, unspecified
K912	Postsurgical malabsorption, not elsewhere classified
M83	Adult osteomalacia
M88	Paget disease of bone [osteitis deformans]
N25	Disorders resulting from impaired renal tubular function
**Excluded ICD-10 codes and labels**	
E231	Drug-induced hypopituitarism
E242	Drug-induced Cushing syndrome
E244	Alcohol-induced pseudo-Cushing syndrome
E273	Drug-induced adrenocortical insufficiency
E343	Short stature, not elsewhere classified
E344	Constitutional tall stature
E350	Disorders of thyroid gland in diseases classified elsewhere
E441	Mild protein-energy malnutrition
E790	Hyperuricaemia without signs of inflammatory arthritis and tophaceous disease
E804	Gilbert syndrome
E883	Tumour lysis syndrome
E890	Postprocedural hypothyroidism
E892	Postprocedural hypoparathyroidism
**OTHER MUSCULOSKELETAL AND JOINT DISEASES**	
**Included ICD-10 codes and labels**	
B902	Sequelae of tuberculosis of bones and joints
M212	Flexion deformity
M213	Wrist or foot drop (acquired)
M214	Flat foot [pes planus] (acquired)
M215	Acquired clawhand, clubhand, clawfoot and clubfoot
M216	Other acquired deformities of ankle and foot
M217	Unequal limb length (acquired)
M218	Other specified acquired deformities of limbs
M219	Acquired deformity of limb, unspecified
M22	Disorders of patella
M23	Internal derangement of knee
M24	Other specific joint derangements
M252	Flail joint
M253	Other instability of joint
M357	Hypermobility syndrome
M61	Calcification and ossification of muscle
M652	Calcific tendinitis
M653	Trigger finger

M654	Radial styloid tenosynovitis [de Quervain]
M700	Chronic crepitant synovitis of hand and wrist
M720	Palmar fascial fibromatosis [Dupuytren]
M722	Plantar fascial fibromatosis
M724	Pseudosarcomatous fibromatosis
M750	Adhesive capsulitis of shoulder
M751	Rotator cuff syndrome
M753	Calcific tendinitis of shoulder
M754	Impingement syndrome of shoulder
M797	Fibromyalgia
M841	Nonunion of fracture [pseudarthrosis]
M89	Other disorders of bone
M91	Juvenile osteochondrosis of hip and pelvis
M93	Other osteochondropathies
M94	Other disorders of cartilage
M96	Postprocedural musculoskeletal disorders, not elsewhere classified
M99	Biomechanical lesions, not elsewhere classified
Q65	Congenital deformities of hip
Q66	Congenital deformities of feet
Q68	Other congenital musculoskeletal deformities
Q71	Reduction defects of upper limb
Q72	Reduction defects of lower limb
Q73	Reduction defects of unspecified limb
Q74	Other congenital malformations of limb(s)
Q77	Osteochondrodysplasia with defects of growth of tubular bones and spine
Q78	Other osteochondrodysplasias
Q796	Ehlers-Danlos syndrome
Q798	Other congenital malformations of musculoskeletal system
Q87	Other specified congenital malformation syndromes affecting multiple systems
S382	Traumatic amputation of external genital organs
S48	Traumatic amputation of shoulder and upper arm
S58	Traumatic amputation of forearm
S68	Traumatic amputation of wrist and hand
S78	Traumatic amputation of hip and thigh
S88	Traumatic amputation of lower leg
S98	Traumatic amputation of ankle and foot
T05	Traumatic amputations involving multiple body regions
T096	Traumatic amputation of trunk, level unspecified
T116	Traumatic amputation of upper limb, level unspecified
T136	Traumatic amputation of lower limb, level unspecified
T147	Crushing injury and traumatic amputation of unspecified body region
T90	Sequelae of injuries of head
T91	Sequelae of injuries of neck and trunk

T92	Sequelae of injuries of upper limb
T93	Sequelae of injuries of lower limb
T94	Sequelae of injuries involving multiple and unspecified body regions
T95	Sequelae of burns, corrosions and frostbite
T96	Sequelae of poisoning by drugs, medicaments and biological substances
T97	Sequelae of toxic effects of substances chiefly nonmedicinal as to source
T98	Sequelae of other and unspecified effects of external causes
Z440	Fitting and adjustment of artificial arm (complete)(partial)
Z441	Fitting and adjustment of artificial leg (complete)(partial)
Z891	Acquired absence of hand and wrist
Z892	Acquired absence of upper limb above wrist
Z893	Acquired absence of both upper limbs [any level]
Z894	Acquired absence of foot and ankle
Z895	Acquired absence of leg at or below knee
Z896	Acquired absence of leg above knee
Z897	Acquired absence of both lower limbs [any level, except toes alone]
Z898	Acquired absence of upper and lower limbs [any level]
Z899	Acquired absence of limb, unspecified
Z946	Bone transplant status
Z966	Presence of orthopaedic joint implants
Z971	Presence of artificial limb (complete)(partial)
**OTHER NEUROLOGICAL DISEASES**	
**Included ICD-10 codes and labels**	
B900	Sequelae of central nervous system tuberculosis
D482	Neoplasm of uncertain or unknown behaviour: Peripheral nerves and autonomic nervous system
G041	Tropical spastic paraplegia
G09	Sequelae of inflammatory diseases of central nervous system
G10	Huntington disease
G11	Hereditary ataxia
G12	Spinal muscular atrophy and related syndromes
G13	Systemic atrophies primarily affecting central nervous system in diseases classified elsewhere
G24	Dystonia
G25	Other extrapyramidal and movement disorders
G26	Extrapyramidal and movement disorders in diseases classified elsewhere
G32	Other degenerative disorders of nervous system in diseases classified elsewhere
G37	Other demyelinating diseases of central nervous system
G51	Facial nerve disorders
G52	Disorders of other cranial nerves
G53	Cranial nerve disorders in diseases classified elsewhere
G70	Myasthenia gravis and other myoneural disorders
G71	Primary disorders of muscles

G723	Periodic paralysis
G724	Inflammatory myopathy, not elsewhere classified
G728	Other specified myopathies
G729	Myopathy, unspecified
G73	Disorders of myoneural junction and muscle in diseases classified elsewhere
G80	Cerebral palsy
G81	Hemiplegia
G82	Paraplegia and tetraplegia
G83	Other paralytic syndromes
G90	Disorders of autonomic nervous system
G91	Hydrocephalus
G938	Other specified disorders of brain
G939	Disorder of brain, unspecified
G95	Other diseases of spinal cord
G99	Other disorders of nervous system in diseases classified elsewhere
M471	Other spondylosis with myelopathy
Q00	Anencephaly and similar malformations
Q01	Encephalocele
Q02	Microcephaly
Q03	Congenital hydrocephalus
Q04	Other congenital malformations of brain
Q05	Spina bifida
Q06	Other congenital malformations of spinal cord
Q07	Other congenital malformations of nervous system
Q760	Spina bifida occulta
**Excluded ICD-10 codes and labels**	
G130	Paraneoplastic neuromyopathy and neuropathy
G131	Other systemic atrophy primarily affecting central nervous system in neoplastic disease
G251	Drug-induced tremor
G254	Drug-induced chorea
G256	Drug-induced tics and other tics of organic origin
G510	Bell palsy
G732	Other myasthenic syndromes in neoplastic disease
G733	Myasthenic syndromes in other diseases classified elsewhere
G734	Myopathy in infectious and parasitic diseases classified elsewhere
G838	Other specified paralytic syndromes
**OTHER PSYCHIATRIC AND BEHAVIORAL DISEASES**	
**Included ICD-10 codes and labels**	
F04	Organic amnesic syndrome, not induced by alcohol and other psychoactive substances
F06	Other mental disorders due to brain damage and dysfunction and to physical disease
F07	Personality and behavioural disorders due to brain disease, damage and dysfunction
F09	Unspecified organic or symptomatic mental disorder
F102	Mental and behavioural disorders due to use of alcohol: Dependence syndrome

F106	Mental and behavioural disorders due to use of alcohol: Amnesic syndrome
F107	Mental and behavioural disorders due to use of alcohol: Residual and late-onset psychotic disorder
F112	Mental and behavioural disorders due to use of opioids: Dependence syndrome
F116	Mental and behavioural disorders due to use of opioids: Amnesic syndrome
F117	Mental and behavioural disorders due to use of opioids: Residual and late-onset psychotic disorder
F122	Mental and behavioural disorders due to use of cannabinoids: Dependence syndrome
F126	Mental and behavioural disorders due to use of cannabinoids: Amnesic syndrome
F127	Mental and behavioural disorders due to use of cannabinoids: Residual and late-onset psychotic disorder
F132	Mental and behavioural disorders due to use of sedatives or hypnotics: Dependence syndrome
F136	Mental and behavioural disorders due to use of sedatives or hypnotics: Amnesic syndrome
F137	Mental and behavioural disorders due to use of sedatives or hypnotics: Residual and late-onset psychotic disorder
F142	Mental and behavioural disorders due to use of cocaine: Dependence syndrome
F146	Mental and behavioural disorders due to use of cocaine: Amnesic syndrome
F147	Mental and behavioural disorders due to use of cocaine: Residual and late-onset psychotic disorder
F152	Mental and behavioural disorders due to use of other stimulants, including caffeine: Dependence syndrome
F156	Mental and behavioural disorders due to use of other stimulants, including caffeine: Amnesic syndrome
F157	Mental and behavioural disorders due to use of other stimulants, including caffeine: Residual and late-onset psychotic disorder
F162	Mental and behavioural disorders due to use of hallucinogens: Dependence syndrome
F166	Mental and behavioural disorders due to use of hallucinogens: Amnesic syndrome
F167	Mental and behavioural disorders due to use of hallucinogens: Residual and late-onset psychotic disorder
F172	Mental and behavioural disorders due to use of tobacco: Dependence syndrome
F176	Mental and behavioural disorders due to use of tobacco: Amnesic syndrome
F177	Mental and behavioural disorders due to use of tobacco: Residual and late-onset psychotic disorder
F182	Mental and behavioural disorders due to use of volatile solvents: Dependence syndrome
F186	Mental and behavioural disorders due to use of volatile solvents: Amnesic syndrome
F187	Mental and behavioural disorders due to use of volatile solvents: Residual and late-onset psychotic disorder
F192	Mental and behavioural disorders due to multiple drug use and use of other psychoactive substances: Dependence syndrome
F196	Mental and behavioural disorders due to multiple drug use and use of other psychoactive substances: Amnesic syndrome
F197	Mental and behavioural disorders due to multiple drug use and use of other psychoactive substances: Residual and late-onset psychotic disorder
F50	Eating disorders
F52	Sexual dysfunction, not caused by organic disorder or disease
F60	Specific personality disorders

F61	Mixed and other personality disorders
F62	Enduring personality changes, not attributable to brain damage and disease
F63	Habit and impulse disorders
F68	Other disorders of adult personality and behaviour
F70	Mild mental retardation
F71	Moderate mental retardation
F72	Severe mental retardation
F73	Profound mental retardation
F78	Other mental retardation
F79	Unspecified mental retardation
F80	Specific developmental disorders of speech and language
F81	Specific developmental disorders of scholastic skills
F82	Specific developmental disorder of motor function
F83	Mixed specific developmental disorders
F84	Pervasive developmental disorders
F88	Other disorders of psychological development
F89	Unspecified disorder of psychological development
F95	Tic disorders
F99	Mental disorder, not otherwise specified
**OTHER RESPIRATORY DISEASES**	
**Included ICD-10 codes and labels**	
B909	Sequelae of respiratory and unspecified tuberculosis
E662	Extreme obesity with alveolar hypoventilation
J60	Coalworker pneumoconiosis
J61	Pneumoconiosis due to asbestos and other mineral fibres
J62	Pneumoconiosis due to dust containing silica
J63	Pneumoconiosis due to other inorganic dusts
J64	Unspecified pneumoconiosis
J65	Pneumoconiosis associated with tuberculosis
J66	Airway disease due to specific organic dust
J67	Hypersensitivity pneumonitis due to organic dust
J684	Chronic respiratory conditions due to chemicals, gases, fumes and vapours
J701	Chronic and other pulmonary manifestations due to radiation
J703	Chronic drug-induced interstitial lung disorders
J704	Drug-induced interstitial lung disorders, unspecified
J84	Other interstitial pulmonary diseases
J92	Pleural plaque
J941	Fibrothorax
J953	Chronic pulmonary insufficiency following surgery
J955	Postprocedural subglottic stenosis
J961	Chronic respiratory failure
J98	Other respiratory disorders
Q33	Congenital malformations of lung

Q34	Other congenital malformations of respiratory system
Z902	Acquired absence of lung [part of]
Z942	Lung transplant status
Z943	Heart and lungs transplant status
Z963	Presence of artificial larynx
**Excluded ICD-10 codes and labels**	
J981	Pulmonary collapse
**OTHER SKIN DISEASES**	
**Included ICD-10 codes and labels**	
L13	Other bullous disorders
L28	Lichen simplex chronicus and prurigo
L301	Dyshidrosis [pompholyx]
L43	Lichen planus
L508	Other urticaria
L581	Chronic radiodermatitis
L85	Other epidermal thickening
Q80	Congenital ichthyosis
Q81	Epidermolysis bullosa
Q821	Xeroderma pigmentosum
Q822	Mastocytosis
Q829	Congenital malformation of skin, unspecified
**Excluded ICD-10 codes and labels**	
L432	Lichenoid drug reaction
**PARKINSON AND PARKINSONISM**	
**Included ICD-10 codes and labels**	
G20	Parkinson disease
G21	Secondary parkinsonism
G22	Parkinsonism in diseases classified elsewhere
G23	Other degenerative diseases of basal ganglia
**Excluded ICD-10 codes and labels**	
G210	Malignant neuroleptic syndrome
**PERIPHERAL NEUROPATHY**	
**Included ICD-10 codes and labels**	
B91	Sequelae of poliomyelitis
G14	Postpolio syndrome
G54	Nerve root and plexus disorders
G55	Nerve root and plexus compressions in diseases classified elsewhere
G56	Mononeuropathies of upper limb
G57	Mononeuropathies of lower limb
G58	Other mononeuropathies
G59	Mononeuropathy in diseases classified elsewhere
G60	Hereditary and idiopathic neuropathy
G628	Other specified polyneuropathies

G629	Polyneuropathy, unspecified
G63	Polyneuropathy in diseases classified elsewhere
M472	Other spondylosis with radiculopathy
M531	Cervicobrachial syndrome
M541	Radiculopathy
**Excluded ICD-10 codes and labels**	
G631	Polyneuropathy in neoplastic disease
**PERIPHERAL VASCULAR DISEASE**	
**Included ICD-10 codes and labels**	
I702	Atherosclerosis of arteries of extremities
I73	Other peripheral vascular diseases
I792	Peripheral angiopathy in diseases classified elsewhere
I798	Other disorders of arteries, arterioles and capillaries in diseases classified elsewhere
**Excluded ICD-10 codes and labels**	
I731	Thromboangiitis obliterans [Buerger]
I738	Other specified peripheral vascular diseases
**PROSTATE DISEASES**	
**Included ICD-10 codes and labels**	
N40	Hyperplasia of prostate
N411	Chronic prostatitis
N418	Other inflammatory diseases of prostate
**SCHIZOPHRENIA AND DELUSIONAL DISEASES**	
**Included ICD-10 codes and labels**	
F20	Schizophrenia
F22	Persistent delusional disorders
F24	Induced delusional disorder
F25	Schizoaffective disorders
F28	Other nonorganic psychotic disorders
**SLEEP DISORDERS**	
**Included ICD-10 codes and labels**	
F510	Nonorganic insomnia
F511	Nonorganic hypersomnia
F512	Nonorganic disorder of the sleep-wake schedule
F513	Sleepwalking [somnambulism]
G47	Sleep disorders
**SOLID NEOPLASMS**	
**Included ICD-10 codes and labels**	
C	Malignant neoplasms
D00	Carcinoma in situ of oral cavity, oesophagus and stomach
D01	Carcinoma in situ of other and unspecified digestive organs
D02	Carcinoma in situ of middle ear and respiratory system
D03	Melanoma in situ
D04	Carcinoma in situ of skin

D05	Carcinoma in situ of breast
D06	Carcinoma in situ of cervix uteri
D07	Carcinoma in situ of other and unspecified genital organs
D09	Carcinoma in situ of other and unspecified sites
D320	Benign neoplasm: Cerebral meninges
D321	Benign neoplasm: Spinal meninges
D329	Benign neoplasm: Meninges, unspecified
D330	Benign neoplasm: Brain, supratentorial
D331	Benign neoplasm: Brain, infratentorial
D332	Benign neoplasm: Brain, unspecified
D333	Benign neoplasm: Cranial nerves
D334	Benign neoplasm: Spinal cord
Q85	Phakomatoses, not elsewhere classified
**Excluded ICD-10 codes and labels**	
C81	Hodgkin lymphoma
C82	Follicular lymphoma
C83	Non-follicular lymphoma
C84	Mature T/NK-cell lymphomas
C85	Other and unspecified types of non-Hodgkin lymphoma
C86	Other specified types of T/NK-cell lymphoma
C88	Malignant immunoproliferative diseases
C90	Multiple myeloma and malignant plasma cell neoplasms
C91	Lymphoid leukaemia
C92	Myeloid leukaemia
C93	Monocytic leukaemia
C94	Other leukaemias of specified cell type
C95	Leukaemia of unspecified cell type
C96	Other and unspecified malignant neoplasms of lymphoid, haematopoietic and related tissue
**THYROID DISEASES**	
**Included ICD-10 codes and labels**	
E00	Congenital iodine-deficiency syndrome
E01	Iodine-deficiency-related thyroid disorders and allied conditions
E02	Subclinical iodine-deficiency hypothyroidism
E03	Other hypothyroidism
E05	Thyrotoxicosis [hyperthyroidism]
E062	Chronic thyroiditis with transient thyrotoxicosis
E063	Autoimmune thyroiditis
E065	Other chronic thyroiditis
E07	Other disorders of thyroid
E350	Disorders of thyroid gland in diseases classified elsewhere
E890	Postprocedural hypothyroidism
**Excluded ICD-10 codes and labels**	
E035	Myxoedema coma

VENOUS AND LYMPHATIC DISEASES	
**Included ICD-10 codes and labels**	
I780	Hereditary haemorrhagic telangiectasia
I83	Varicose veins of lower extremities
I87	Other disorders of veins
I89	Other noninfective disorders of lymphatic vessels and lymph nodes
I972	Postmastectomy lymphoedema syndrome
Q820	Hereditary lymphoedema

Supplementary table 4. Percentage (**row percentages**) of participants moving from one cluster to another between baseline and 6 years.

					ROW PERCENTAGE				
	6-YEAR CLUSTERS								
	Heart and vasc.	Heart and cogn.	Neurops. and resp.	Eye	MSK, resp. and immun.	Unspecific	Death	Dropout	Total
BASELINE CLUSTERS									
Psychiatric and respiratory, n	2	7	51	10	11	18	35	25	159
%	1,26	4,4	32,08	6,29	6,92	11,32	22,01	15,72	100
Heart, n	15	63	7	4	3	4	146	35	277
%	5,42	22,74	2,53	1,44	1,08	1,44	52,71	12,64	100
Eye and Cancer, n	7	22	27	78	6	17	105	43	305
%	2,3	7,21	8,85	25,57	1,97	5,57	34,43	14,1	100
Cognitive and sensory, n	3	3	33	9	2	4	233	19	306
%	0,98	0,98	10,78	2,94	0,65	1,31	76,14	6,21	100
RESP-MSKRespiratory and MSK	12	13	60	38	109	68	91	65	456
%	2,63	2,85	13,16	8,33	23,9	14,91	19,96	14,25	100
Unspecific, n	37	44	87	88	107	647	203	215	1.428
%	2,59	3,08	6,09	6,16	7,49	45,31	14,22	15,06	100
Total, n	76	152	265	227	238	758	813	402	2.931
%	2,59	5,19	9,04	7,74	8,12	25,86	27,74	13,72	100

Supplementary table 5. Percentage (**column percentages**) of participants moving from one cluster to another between baseline and 6 years.

					COLUMN PERCENTAGE				
	6-YEAR CLUSTERS								
	Heart and vasc.	Heart and cogn.	Neurops. and resp.	Eye	MSK, resp. and immun.	Unspecific	Death	Dropout	Total
BASELINE CLUSTERS									
Psychiatric and respiratory, n	2	7	51	10	11	18	35	25	159
%	2,63	4,61	19,25	4,41	4,62	2,37	4,31	6,22	5,42
Heart, n	15	63	7	4	3	4	146	35	277
%	19,74	41,45	2,64	1,76	1,26	0,53	17,96	8,71	9,45
Eye and Cancer, n	7	22	27	78	6	17	105	43	305
%	9,21	14,47	10,19	34,36	2,52	2,24	12,92	10,7	10,41
Cognitive and sensory, n	3	3	33	9	2	4	233	19	306
%	3,95	1,97	12,45	3,96	0,84	0,53	28,66	4,73	10,44
RESP-MSKRespiratory and MSK	12	13	60	38	109	68	91	65	456
%	15,79	8,55	22,64	16,74	45,8	8,97	11,19	16,17	15,56
Unspecific, n	37	44	87	88	107	647	203	215	1.428
%	48,68	28,95	32,83	38,77	44,96	85,36	24,97	53,48	48,72
Total, n	76	152	265	227	238	758	813	402	2.931
%	100	100	100	100	100	100	100	100	100

Supplementary table 6. Percentage (**row percentages**) of participants moving from one cluster to another between 6 and 12 years.

						ROW PERCENTAGE			
	12-YEAR CLUSTERS								
	Vascular	Cardiometabolic	Respiratory	Neuropsyc	Eye and MSK	Unspecific	Death	Dropout	Total
6-YEAR CLUSTERS									
Heart and vascular, n	19	4	0	1	1	2	37	12	76
%	25	5,26	0	1,32	1,32	2,63	48,68	15,79	100
Heart and cogn., n	5	31	2	4	3	0	93	14	152
%	3,29	20,39	1,32	2,63	1,97	0	61,18	9,21	100
Neurops. and resp, n	4	5	19	31	12	22	143	29	265
%	1,51	1,89	7,17	11,7	4,53	8,3	53,96	10,94	100
Eye, n	10	12	5	42	52	4	79	23	227
%	4,41	5,29	2,2	18,5	22,91	1,76	34,8	10,13	100
MSK, resp and imm, n	12	7	73	18	33	34	32	29	238
%	5,04	2,94	30,67	7,56	13,87	14,29	13,45	12,18	100
Unspecific, n	13	57	19	24	99	337	93	116	758
%	1,72	7,52	2,51	3,17	13,06	44,46	12,27	15,3	100
Total, n	63	116	118	120	200	399	477	223	1.716
%	3,67	6,76	6,88	6,99	11,66	23,25	27,8	13	100

Supplementary table 7. Percentage (**column percentages**) of participants moving from one cluster to another between 6 and 12 years.

						COLUMN PERCENTAGE			
	12-YEAR CLUSTERS								
	Vascular	Cardiometabolic	Respiratory	Neuropsyc	Eye and MSK	Unspecific	Death	Dropout	Total
6-YEAR CLUSTERS									
Heart and vascular, n	19	4	0	1	1	2	37	12	76
%	30,16	3,45	0	0,83	0,5	0,5	7,76	5,38	4,43
Heart and cogn., n	5	31	2	4	3	0	93	14	152
%	7,94	26,72	1,69	3,33	1,5	0	19,5	6,28	8,86
Neurops. and resp, n	4	5	19	31	12	22	143	29	265
%	6,35	4,31	16,1	25,83	6	5,51	29,98	13	15,44
Eye, n	10	12	5	42	52	4	79	23	227
%	15,87	10,34	4,24	35	26	1	16,56	10,31	13,23
MSK, resp and imm, n	12	7	73	18	33	34	32	29	238
%	19,05	6,03	61,86	15	16,5	8,52	6,71	13	13,87
Unspecific, n	13	57	19	24	99	337	93	116	758
%	20,63	49,14	16,1	20	49,5	84,46	19,5	52,02	44,17
Total, n	63	116	118	120	200	399	477	223	1.716
%	100	100	100	100	100	100	100	100	100

Validation indices at baseline

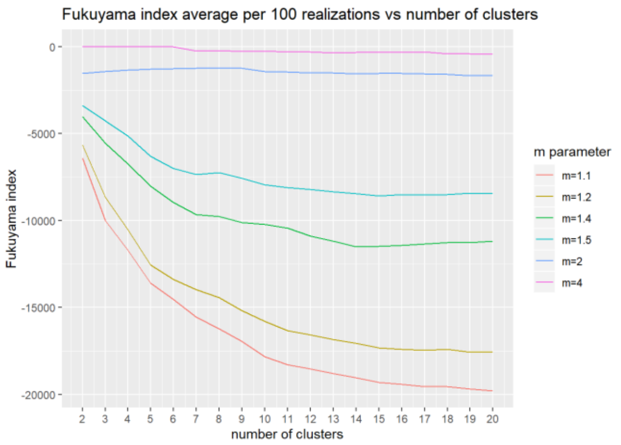

Supplementary figure 1. Fukuyama index across increasing number of clusters at baseline.

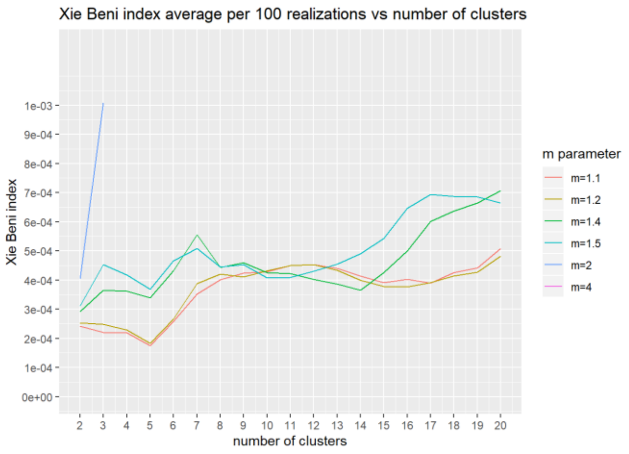

Supplementary figure 2. Xie Beni index across increasing number of clusters at baseline.

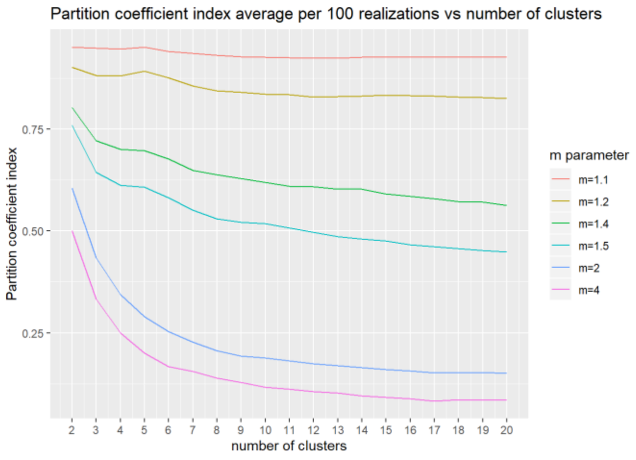

Supplementary figure 3. Partition coefficient index across increasing number of clusters at baseline.

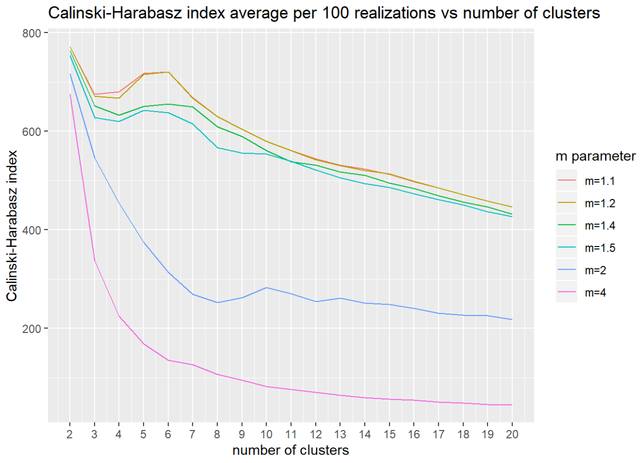

Supplementary figure 4. Calinski-Harabasz index across increasing number of clusters at baseline.

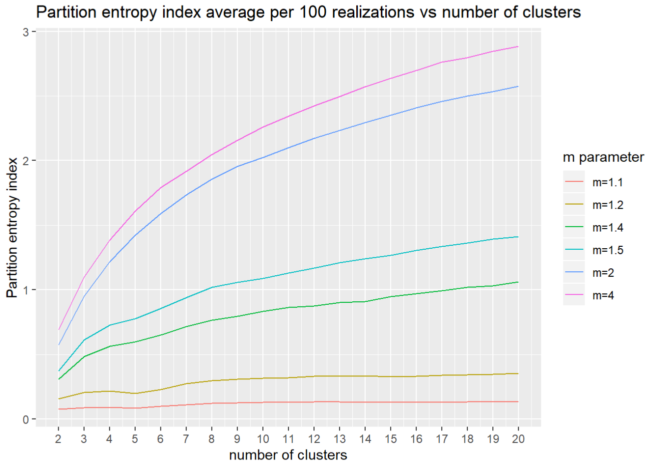

Supplementary figure 5. Partition entropy index across increasing number of clusters at baseline.

Validation indices at 6 years

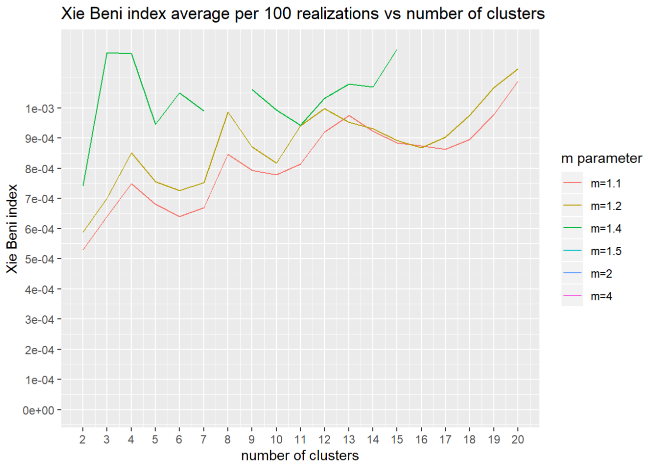

Supplementary figure 6. Xie Beni index across increasing number of clusters at 6 years.

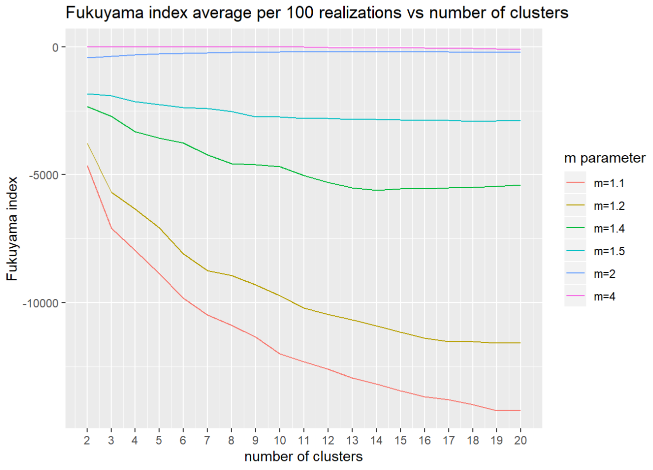

Supplementary figure 7. Fukuyama index across increasing number of clusters at 6 years.

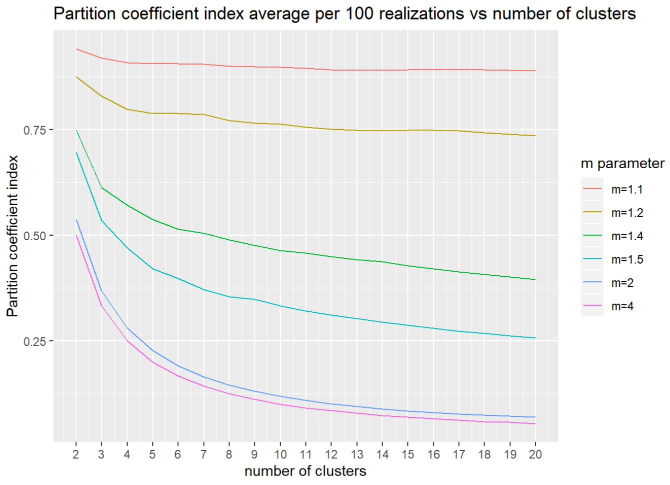

Supplementary figure 8. Partition coefficient index across increasing number of clusters at 6 years.

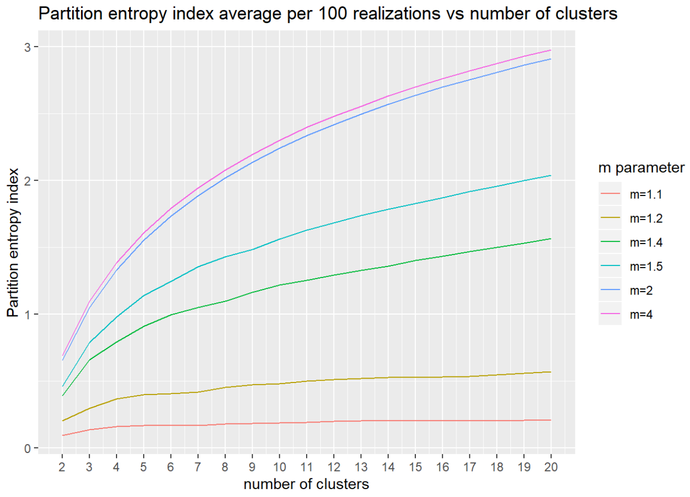

Supplementary figure 9. Partition entropy index across increasing number of clusters at 6 years.

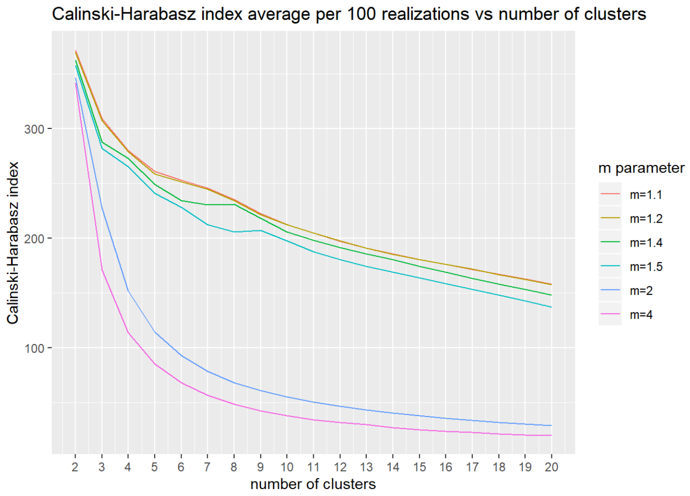

Supplementary figure 10. Calinski-Harabasz index across increasing number of clusters at 6 years.

Validation indices at 12 years

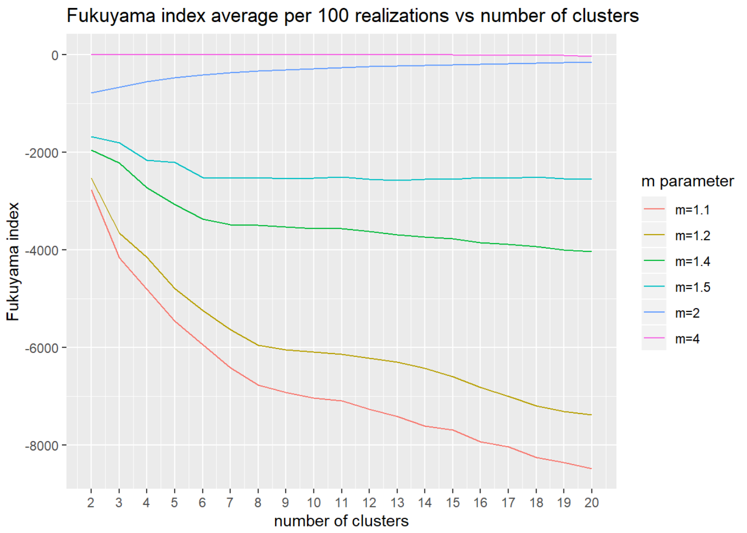

Supplementary figure 11. Fukuyama index across increasing number of clusters at 12 years.

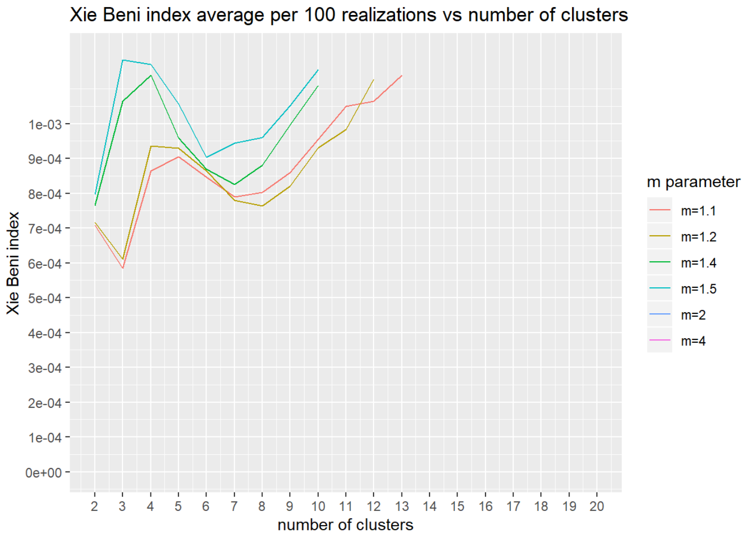

Supplementary figure 12. Xie Beni index across increasing number of clusters at 12 years.

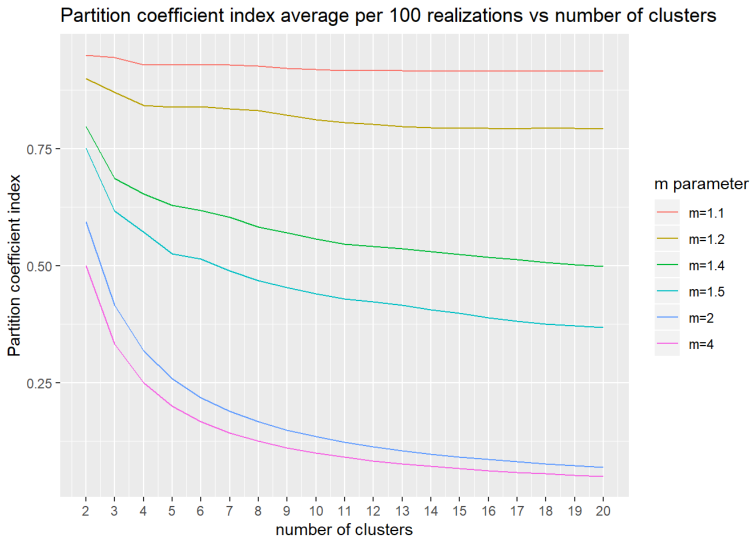

Supplementary figure 13. Partition coefficient index across increasing number of clusters at 12 years.

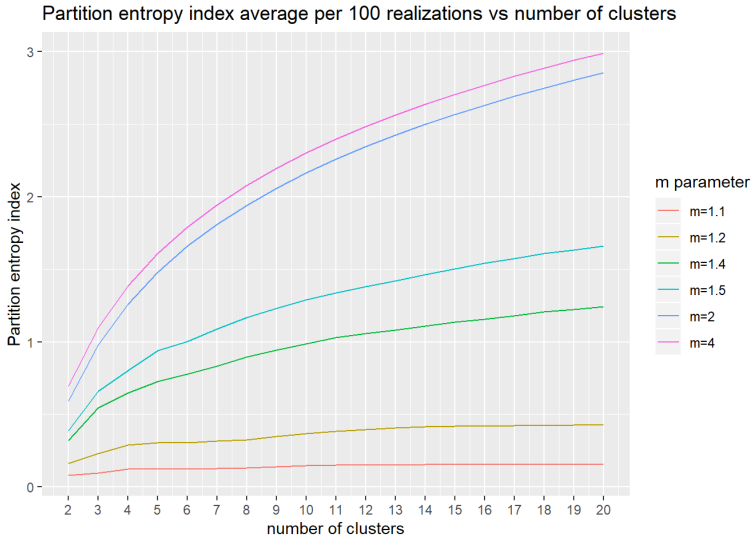

Supplementary figure 14. Partition entropy index across increasing number of clusters at 12 years.

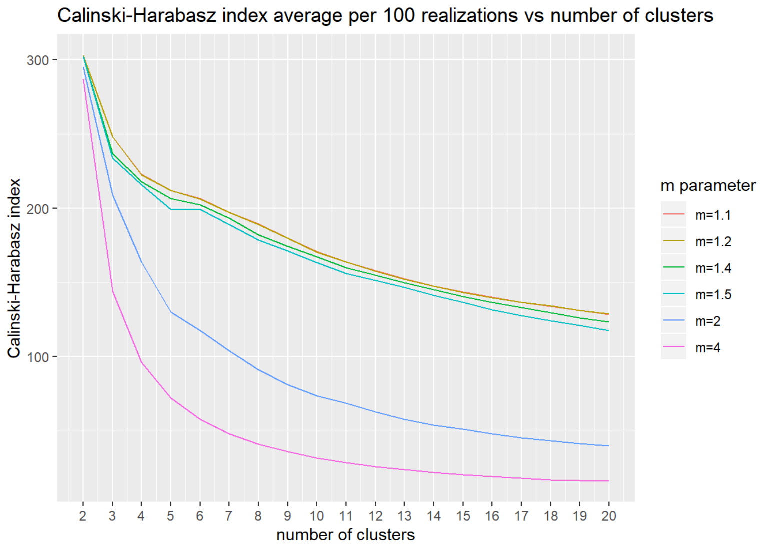

Supplementary figure 15. Calinski-Harabasz index across increasing number of clusters at 12 years.
