# Supplementary material for: Twelve-year clinical trajectories of multimorbidity in a population of older adults
Source: Nat Commun. 2020 Jun 26;11:3223. doi: 10.1038/s41467-020-16780-x (PMC7320143; doi:10.1038/s41467-020-16780-x)
Supplement: Supplementary file 3 — Reporting Summary [file 41467_2020_16780_MOESM3_ESM.pdf]

nature research

Corresponding author(s): Davide L Vetrano

Last updated by author(s): Apr 10, 2020

Reporting Summary

Nature Research wishes to improve the reproducibility of the work that we publish. This form provides structure for consistency and transparency in reporting. For further information on Nature Research policies, see Authors & Referees and the Editorial Policy Checklist .

Statistics

For all statistical analyses, confirm that the following items are present in the figure legend, table legend, main text, or Methods section.

n/a	Confirmed	
☐	☒	The exact sample size (*n*) for each experimental group/condition, given as a discrete number and unit of measurement
☒	☐	A statement on whether measurements were taken from distinct samples or whether the same sample was measured repeatedly
☐	☒	The statistical test(s) used AND whether they are one- or two-sided<br>*Only common tests should be described solely by name; describe more complex techniques in the Methods section.*
☐	☒	A description of all covariates tested
☐	☒	A description of any assumptions or corrections, such as tests of normality and adjustment for multiple comparisons
☐	☒	A full description of the statistical parameters including central tendency (e.g. means) or other basic estimates (e.g. regression coefficient) AND variation (e.g. standard deviation) or associated estimates of uncertainty (e.g. confidence intervals)
☒	☐	For null hypothesis testing, the test statistic (e.g. *F*, *t*, *r*) with confidence intervals, effect sizes, degrees of freedom and *P* value noted<br>*Give P values as exact values whenever suitable.*
☒	☐	For Bayesian analysis, information on the choice of priors and Markov chain Monte Carlo settings
☒	☐	For hierarchical and complex designs, identification of the appropriate level for tests and full reporting of outcomes
☐	☒	Estimates of effect sizes (e.g. Cohen's *d*, Pearson's *r*), indicating how they were calculated

Our web collection on statistics for biologists contains articles on many of the points above.

Software and code

Policy information about availability of computer code

Data collection	Data were collected through dedicated forms. Information is later electronically read and stored in Stata. The data quality check is regularly carried out following specific and rigorous protocols (se: https://www.snac-k.se/). Stata and R dataset files have been eventually produced to carry out the analyses.
Data analysis	Standard descriptive statistics (mean, standard deviation, count and percentage) have been carried out to report the sample characteristics at baseline, 6 andd 12 years. C-mean cluster analyses have been carried out to identify homogeneous groups of participants based on their underlying disease patterns. Logistic regression models have been employed to test the association between the clusters and death. Statistical analyses were performed using R 3.5.1 and Stata 15.0

For manuscripts utilizing custom algorithms or software that are central to the research but not yet described in published literature, software must be made available to editors/reviewers. We strongly encourage code deposition in a community repository (e.g. GitHub). See the Nature Research guidelines for submitting code & software for further information.

Data

Policy information about availability of data

All manuscripts must include a data availability statement. This statement should provide the following information, where applicable:

- Accession codes, unique identifiers, or web links for publicly available datasets
- A list of figures that have associated raw data
- A description of any restrictions on data availability

Data are from the SNAC-K project, a population-based study on aging and dementia (http://www.snac-k.se/). Access to these original data is available to the research community upon approval by the SNAC-K data management and maintenance committee. Applications for accessing these data can be submitted to Maria Wahlberg (Maria.Wahlberg@ki.se) at the Aging Research Center, Karolinska Institutet.

Field-specific reporting

Please select the one below that is the best fit for your research. If you are not sure, read the appropriate sections before making your selection.

- [x] Life sciences
- [ ] Behavioural & social sciences
- [ ] Ecological, evolutionary & environmental sciences

For a reference copy of the document with all sections, see nature.com/documents/nr-reporting-summary-flat.pdf

Life sciences study design

All studies must disclose on these points even when the disclosure is negative.

Sample size	No sample size calculations have been carried out. The prevalence of multimorbidity in the sample population (90%) and the 12-year follow up , as shown in previous studies (https://www.snac-k.se/) provide enough power to test the hypotheses that 1) different multimorbidity clusters can be identified with reliable and stable statistical parameters and that 2) such clusters are differently associated to death. The present study is based on the population-based study SNAC-K, which includes at baseline 3363 individuals aged 60+. SNAC-K is one of the biggest population-based studies on aging, which size allows to study most of the age-related conditions with decent statistical power, including multimorbidity. In SNAC-K, multimorbidity has a prevalence as high as 88.6%. For the present study 2931 participants with at least two chronic diseases have been included.
Data exclusions	Being the aim of the study to analyze multimorbidity clusters, 432 subjects with zero or only one diseases have been excluded from the initial SNAC-K population. In order to identify clusters of individuals with multimorbidity, at least two diseases should be displayed by each participants. This is in line with what a priori established by the study's researchers.
Replication	The rigorous disease clinical assessment, the representativeness of the sample population (response rate 73%, among the highest in this field), and the use of clear statistical methods ease the replicability of the present study.
Randomization	This is an observational study which participants have been randomly selected by the population 60+ resident in the Kungsholmen area. Municipality census data have been used to randomize the invitation to participate to the study.
Blinding	No blinded procedures are requested for this observational population-based study.

Reporting for specific materials, systems and methods

We require information from authors about some types of materials, experimental systems and methods used in many studies. Here, indicate whether each material, system or method listed is relevant to your study. If you are not sure if a list item applies to your research, read the appropriate section before selecting a response.

Materials & experimental systems

n/a	Involved in the study	
x		Antibodies
x		Eukaryotic cell lines
x		Palaeontology
x		Animals and other organisms
	x	Human research participants
x		Clinical data

Methods

n/a	Involved in the study	
x		ChIP-seq
x		Flow cytometry
x		MRI-based neuroimaging

Human research participants

Policy information about studies involving human research participants

Population characteristics	At baseline, study participants' mean age was 76.1 ± 11.0 and 1951 (66.6%) were female. On average, participants suffered from 4.5 diseases and were on average on 4.4 medications. The number of people dropping from the study was 402 (13.7%) in the first 6 years and 223 (13.0%) between 6 and 12 years.
Recruitment	The study population consists of adults ≥60 years living in the community or in institutions, from the Kungsholmen district of Stockholm, Sweden. A random sample of 11 age cohorts born between 1892 and 1939 (the youngest and oldest age cohorts were oversampled) was invited to participate in the study. People who agreed to participate were evaluated for the first time between 2001 and 2004. Participants who were <78 years of age were then followed up every six years and participants ≥78 years every three years. The present study is based on data collected at baseline, six years, and 12 years. At baseline, 3363 people were examined (participation rate 73%). Non-participants were older, more likely females, and more likely institutionalized. As a consequence, non-participants may have presented a higher disease burden. A participation rate of 73% remains among the highest reported in population-based studies involving older people. The participation rate <100% represent a potential source of selection bias.
Ethics oversight	The study was approved by the Regional Ethics Review Board in Stockholm

Note that full information on the approval of the study protocol must also be provided in the manuscript.
